# *Notes from the Field:* Diarrhea and Acute Respiratory Infection, Oral Cholera Vaccination Coverage, and Care-Seeking Behaviors of Rohingya Refugees — Cox’s Bazar, Bangladesh, October–November 2017

**DOI:** 10.15585/mmwr.mm6718a6

**Published:** 2018-05-11

**Authors:** Aimee Summers, Alexa Humphreys, Eva Leidman, Leonie Toroitich Van Mil, Caroline Wilkinson, Anuradhha Narayan, MALD, Mohammad Lalan Miah, Blanche Greene Cramer, Oleg Bilukha

**Affiliations:** ^1^Division of Global Health Protection, Center for Global Health, CDC; ^2^Action Against Hunger, New York City, New York ^3^United Nations High Commissioner for Refugees, Geneva Switzerland; ^4^United Nations Children’s Fund, New York City, New York.

Violence in the Rakhine State of Myanmar, which began on August 25, 2017, prompted mass displacement of Rohingya to the bordering district of Cox’s Bazar, Bangladesh. Joining the nearly 213,000 Rohingya already in the region, an estimated 45,000 persons settled in two preexisting refugee camps, Nayapara and Kutupalong, and nearly 550,000 into new makeshift settlements (*1*). Mass violence and displacement, accompanied by malnutrition, overcrowding, poor hygiene, and lack of access to safe water and health care increase the vulnerability of children to infectious diseases, including pneumonia and diarrhea (*2*).

To prevent an outbreak of cholera, which is endemic in Bangladesh, a fixed-site, mass oral cholera vaccination (OCV) campaign targeting all persons aged ≥1 year was conducted among Rohingya refugees during October 10–18, with a follow-up campaign targeting children aged 1–4 years November 4–9 (*3*). Three cross-sectional population-representative household surveys were conducted in Kutupalong (October 22–28), makeshift settlements (October 29–November 20), and Nayapara (November 20–27). Sampling frames included all households in each area regardless of whether they were registered with the Office of the United Nations High Commissioner for Refugees (UNHCR). Registered refugees had access to a full spectrum of services provided by UNHCR, including health care, food vouchers, and nutrition treatment programs. In Kutupalong and Nayapara, households were selected using simple random sampling. Camps were enumerated the week preceding data collection. Because of the large population residing in the makeshift settlements, households in these sites were selected using multistage cluster sampling; the Inter Sector Coordination Group (a coordination body consisting of international and domestic agencies responding to the refugee crisis and led by the International Organization for Migration) provided block populations. 

The surveys assessed diarrhea and acute respiratory infection (ARI)–associated morbidity in children aged 6–59 months and care-seeking behaviors of parents or caregivers for those children with diarrhea or ARI-associated morbidity, as well as receipt of at least one OCV dose in all persons aged ≥1 year. A 2-week cumulative incidence of diarrhea was ascertained by asking caregivers whether the child had three or more loose stools within the 2 weeks preceding the survey; ARI was defined as having cough with rapid breathing or difficulty breathing and a fever within the 2 weeks preceding the survey. Caregivers reporting morbidity were asked separately for each condition whether the child had been taken for treatment at a clinic or hospital managed by the Bangladesh government or a humanitarian organization (the formal health system), a traditional healer, a local pharmacy, another location/provider, or were not taken for treatment. P values were calculated using two-proportion t-tests to assess differences between registered and unregistered refugees in Kutupalong and Nayapara camps and old arrivals and new arrivals in makeshift settlements. Analysis of data from makeshift settlements accounted for the multistage cluster survey design.

Two-week cumulative incidence of ARI (50.3%–57.7%) and diarrhea (34.3%–41.3%) were high in all settings (Table). In Kutupalong Camp, unregistered refugees had significantly higher diarrhea-associated morbidity (p<0.001), and ARI-associated morbidity (p = 0.002) than did registered refugees. In Nayapara Camp, only diarrhea-associated morbidity was significantly higher among unregistered refugees than among registered refugees (p = 0.004). A large proportion of parents or caregivers sought health care for their children outside the formal health care system or did not seek care for their children with ARI (27.4%–44.2%) or diarrhea (36.4%–49.6%), even among registered refugees. Coverage with at least 1 OCV dose was high in Nayapara and makeshift settlements (>81%), however, coverage in Kutupalong was lower (72.6% and 78.9% in children aged 1–4 years and persons aged ≥5 years, respectively) because of the low coverage among unregistered refugees (Table). OCV coverage within camps was similar among children aged 1–4 years and persons aged ≥5 years in all groups (overall, registered, and unregistered refugees) except in Nayapara where coverage among children aged 1–4 years was approximately 10 percentage points higher than that among persons aged ≥5 years in all groups.

**TABLE:** Cumulative 2-week incidence of acute respiratory infections (ARI) and diarrhea, percentage of caregivers seeking care for Rohingya children, and oral cholera vaccine (OCV) coverage in Kutupalong Refugee Camp, Nayapara Refugee Camp, and makeshift settlements — Cox’s Bazar, Bangladesh, October–November, 2017

Location	Overall		Unregistered		Registered	
Population/Health indicator	No.	% (95% CI)	No.	% (95% CI)	No.	% (95% CI)
Kutupalong Refugee Camp*						
Children aged 6–59 mos (total no.)	**309**	**—**	**141**	**—**	**161**	**—**
ARI	172	55.7 (50.1–61.1)	92	65.3 (57.0–72.7)	77	47.8 (40.2–55.6)
ARI treatment						
Formal health system	96	55.8 (48.2–63.1)	48	52.2 (41.9–62.3)	45	58.4 (47.1–69.0)
Other^§^	51	29.7 (23.3–37.0)	26	28.3 (19.9–38.4)	25	32.5 (22.9–43.8)
None	25	14.5 (10.0–20.7)	18	19.6 (12.6–29.0)	7	9.1 (4.4–18.0)
Diarrhea	125	40.5 (35.1–46.1)	73	51.8 (43.5–59.9)	49	30.4 (23.8–38.0)
Diarrhea treatment						
Formal health system	63	50.4 (41.6–59.2)	33	45.2 (34.1–56.8)	27	55.1 (41.0–68.5)
Other^§^	43	34.4 (26.5–43.3)	24	32.9 (23.0–44.6)	19	38.8 (26.1–53.2)
None	19	15.2 (9.9–22.7)	16	21.9 (13.8–33.0)	3	6.1 (2.0–17.6)
Receipt of OCV						
Children aged 1–4 yrs (no.)	277	**—**	126	**—**	144	**—**
Received OCV^¶,^**	201	72.6 (67.0–77.5)	61	48.4 (39.8–57.2)	135	93.8 (88.4–96.7)
Persons aged ≥5 yrs (no.)	1,847	**—**	581	**—**	1,226	**—**
Received OCV^¶,^**	1,458	78.9 (77.0–80.7)	288	49.6 (45.5–53.6)	1,135	92.6 (91.0–93.9)
Nayapara Refugee Camp*						
Children aged 6–59 mos (total no.)	408	**—**	199	**—**	186	**—**
ARI	205	50.3 (45.4–55.1)	100	50.3 (43.3–57.2)	94	50.5 (43.4–57.7)
ARI treatment						
Formal health system	149	72.7 (66.1–78.4)	72	72.0 (62.3–80.0)	72	76.6 (66.9–84.1)
Other^§^	27	13.2 (9.2–18.6)	10	10.0 (5.4–17.7)	14	14.9 (9.0–23.7)
None	29	14.2 (10.0–19.7)	18	18.0 (11.6–26.9)	8	8.5 (4.3–16.2)
Diarrhea	140	34.3 (29.9–39.1)	81	40.7 (34.1–47.7)	50	26.9 (21.0–33.7)
Diarrhea treatment						
Formal health system	89	63.6 (55.2–71.2)	53	65.4 (54.3–75.1)	30	60.0 (45.8–72.7)
Other^§^	28	20.0 (14.1–27.6)	13	16.1 (9.5–25.9)	13	26.0 (15.6–40.0)
None	23	16.4 (11.1–23.6)	15	18.5 (11.4–28.6)	7	14.0 (6.8–26.8)
Receipt of OCV						
Children aged 1–4 yrs (total no.)	373	**—**	182	**—**	170	**—**
Received OCV^¶,^**	355	95.2 (92.5–97.0)	167	91.8 (86.8–95.0)	168	98.8 (95.4–99.7)
Persons aged ≥5 yrs (total no.)	2,629	**—**	976	**—**	168	**—**
Received OCV^¶,^**	2,265	86.2 (84.8–87.4)	796	81.6 (79.0–83.9)	1,363	88.8 (87.1–90.3)
**New makeshift settlements^†^**						
Children aged 6–59 mos (total no.)	1,110	**—**	954	**—**	145	**—**
ARI	640	57.7 (52.8–62.4)	547	57.3 (52.2–62.4)	83	57.2 (45.6–68.1)
ARI treatment						
Formal health system	415	64.8 (58.3–70.9)	355	64.9 (57.7–71.5)	56	67.5 (55.1–77.8)
Other^§^	105	16.4 (12.9–20.6)	86	15.7 (12.0–20.4)	16	19.3 (11.7–30.1)
None	120	18.8 (14.2–24.3)	106	19.4 (14.3–25.8)	11	13.3 (8.5–20.0)
Diarrhea	458	41.3 (36.5–46.2)	399	41.8 (36.8–47.0)	50	34.5 (22.4–49.0)
Diarrhea treatment						
Formal health system	261	57.0 (47.7–65.8)	239	59.9 (49.5–69.5)	21	42.0 (30.6–54.3)
Other^§^	141	30.8 (22.5–40.6)	116	29.1 (20.1–40.1)	18	36.0 (23.4–50.9)
None	56	12.2 (8.5–17.2)	44	11.0 (7.7–15.6)	11	22.0 (11.0–39.1)
Receipt of OCV						
Children aged 1–4 yrs (total no.)	974	**—**	832	**—**	134	**—**
Received OCV^¶,^**	886	91.0 (86.2–94.2)	747	89.8 (84.3–93.5)	131	97.8 (91.0–99.5)
Persons aged ≥5 yrs (total no.)	4,897	**—**	4,180	**—**	670	**—**
Received OCV^¶,^**	4,309	88.0 (83.4–91.4)	3,612	86.4 (81.2–90.3)	670	97.1 (92.1–99.0)

**Abbreviation:** CI = confidence interval.

* Overall results include registered refugees, unregistered refugees arriving before August 25, 2017, and unregistered refugees arriving after August 25, 2017; disaggregated analysis excludes unregistered refugees arriving before August 25, 2017.

^†^ Overall results include registered refugees, old arrivals (unregistered refugees arriving before August 25, 2017), and new arrivals (unregistered refugees arriving after August 25, 2017); disaggregated analysis excludes registered refugees.

^§^ Other treatment includes all those outside of formal health clinics and hospitals including community or traditional healers, local pharmacies, and other not specified.

^¶^ OCV coverage ascertained by recall.

** First round of the OCV campaign occurred October 10–18, 2017, targeting all persons aged ≥1 year; second round of the OCV campaign occurred November 4–9, 2017, targeting children aged 1–4 years; these coverage data include receipt of 1 dose of OCV for persons aged ≥5 years and at least 1 dose of OCV for children aged 1–4 years.

Outbreaks of infectious diseases are common in sites like the assessed camps, which are densely populated and have limited infrastructure and sanitation (*2*), and high cumulative incidence of ARI and diarrhea were observed in this survey population. Coverage with at least 1 dose of OCV was high in all settings except among unregistered refugees in Kutupalong Camp, which might be a consequence of their arrival in the midst of the campaign; survey respondents were not asked about reasons for nonvaccination. In a study of the protective efficacy of OCV in an area where cholera is highly endemic, a single dose of OCV provided 40% protection 6 months after vaccination among persons vaccinated at age 1 year or older (*4*). Thus, at least 2 OCV doses are recommended, depending on the vaccine used, particularly among younger children (*5*).

In response to the high ARI-associated morbidity and an ongoing diphtheria outbreak, a mass vaccination campaign with pentavalent (protecting against diphtheria, pertussis, tetanus, *Haemophilus influenzae* type B, and hepatitis B) and pneumococcal conjugate vaccines was conducted in mid-December, targeting children aged 6 weeks–6 years (*6*). Measures to improve access to safe water and sanitation facilities and hygiene promotion, with an emphasis on handwashing, combined with humanitarian action focused on strengthening the World Health Organization’s Expanded Programme on Immunization for all Rohingya refugees would help reduce the incidence of ARI and diarrheal disease (*7*). In addition, promotion of established health care facilities by community outreach programs can help to ensure safe and appropriate treatment.
